# Germination, physio-anatomical behavior, and productivity of wheat plants irrigated with magnetically treated seawater

**DOI:** 10.3389/fpls.2022.923872

**Published:** 2022-08-17

**Authors:** Dalia Abdel-Fattah H. Selim, Muhammad Zayed, Maha M. E. Ali, Heba S. Eldesouky, Mercedes Bonfill, Amira M. El-Tahan, Omar M. Ibrahim, Mohamed T. El-Saadony, Khaled A. El-Tarabily, Synan F. AbuQamar, Samira Elokkiah

**Affiliations:** ^1^Department of Agricultural Botany, Faculty of Agricultural, Menoufia University, Shibin El-Kom, Egypt; ^2^Department of Botany and Microbiology, Menoufa University, Shebin El-Kom, Egypt; ^3^Department of Soils and Water, Faculty of Agriculture, Benha University, Toukh, Egypt; ^4^Department of Botany, Faculty of Agriculture, Benha University, Toukh, Egypt; ^5^Department of Plant Physiology, Faculty of Pharmacy, University of Barcelona, Barcelona, Spain; ^6^Department of Plant Production, Arid Lands Cultivation Research Institute, The City of Scientific Research and Technological Applications, SRTA-City, Alexandria, Egypt; ^7^Department of Agricultural Microbiology, Faculty of Agriculture, Zagazig University, Zagazig, Egypt; ^8^Department of Biology, College of Science, United Arab Emirates University, Al-Ain, United Arab Emirates; ^9^Khalifa Center for Genetic Engineering and Biotechnology, United Arab Emirates University, Al-Ain, United Arab Emirates; ^10^Harry Butler Institute, Murdoch University, Murdoch, WA, Australia; ^11^Department of Agricultural Botany, Faculty of Agriculture, Kafrelsheikh University, Kafrelsheikh, Egypt

**Keywords:** chemical constituents, growth, leaf blade and stem structure, magnetic field, seawater, water relations

## Abstract

Salinity is an abiotic stress that reduces the seed germination and productivity of wheat. The objective of this study was to assess the impact of irrigation with magnetically treated seawater on the germination, growth, certain physiological and anatomical parameters, and production attributes of wheat (*Triticum aestivum* L.) cv. Sakha 93 plants. Experiments were conducted in the Experimental Farm of the Faculty of Agriculture, Menoufia University, Egypt, during two consecutive winter seasons. Pot experiments involved ten treatments with non-magnetized and magnetized water with various degrees of salinity. Plant samples were taken 95 days after sowing. Irrigation with magnetically treated seawater was found to have beneficial effects on plant growth, water relations, biochemical characteristics, and yield components compared with untreated plants. The germination of wheat seeds increased 13% when treated with magnetic seawater. On the yield scale, the spike length was increased by 40% in season one, and 82% in season two when compared to the control, while the weight of 100 grains increased by 148% and 171%, in each season, respectively, when treated with magnetic water. The anatomical leaf and stem parameters of the plants were markedly improved by watering with magnetically treated seawater at 10 dS m^−1^ compared to the control. However, the leaf water deficit, transpiration rate, and abscisic acid content in the plant shoots decreased significantly (*p* < 0.05). The use of magnetically treated seawater of up to 7.5 dS m^−1^, instead of tap water, is recommended due to benefits to germination and seedling parameters, growth, yield, and physiological, chemical, and anatomical characteristics. In conclusion, magnetic treatment of seawater improved germination performance, growth, and yield of wheat under saline conditions.

## Introduction

Wheat (*Triticum aestivum* L.) is the most important strategic crop in Egypt, and its cultivation area reached 3.8 million acres during the winter growing season of 2018–19, with an entire national output of approximately 9 million tons (FAOSTAT, 2018). Wheat grain has high protein and carbohydrate content, and wheat straw is used as animal feed (Selim et al., 2019).

Water scarcity and drought are increasingly significant environmental challenges impacting both plant growth and human uses. Alternative water sources, such as recycled water, brackish water, seawater, and storm water, have been used for irrigation in various parts of the world and for a variety of purposes, including irrigating agricultural crops (Tyagi et al., 2005). In recent decades, salinity has received more attention, especially in arid and semi-arid areas where there is a serious problem of water shortages allocated for agricultural irrigation (Nerd and Pasternak, 1992). Consequently, to deal with the problem of water scarcity, food and feed crops have been irrigated with saline water. Unfortunately, salt can cause reactive oxygen species (ROS) to form, which can harm membranes and macro molecules (Parvanova et al., 2004).

Plants are harmed by salinity primarily as a result of the osmotic impact and specific ion toxicity (Ferrante et al., 2011). Additionally, salt stress decreases total dry matter (DM) and relative water content (RWC) while increasing proline (Pro) accumulation, enzyme activity, and electrolyte leakage (Tuna et al., 2008). To counteract the damaging effects of salt, plants produce Pro, carbohydrates, and hormones; as well as attracting ions which help the plant’s cells to regulate their osmotic balance and conserve water (Munns, 2002).

Magnetized fields have been found to change the properties of tap water, increasing solubility, pH, viscosity, electrical conductivity (EC), and refractive index, while lowering surface tensions and salt levels in the soil (Takatshinko, 1997). These changes occur by increasing the number of hydrogen bonds through the destruction of larger clusters and the generation of smaller clusters, which allows them to pass through plasma membranes more easily (Wang et al., 2013). Under normal and saline conditions, magnetic water treatments have been demonstrated to enhance germination rates, growth rates, leaf areas, water content, chloroplast levels, enzyme activity, nutrient absorption rates, chemical content, anatomical measurements, and overall yields of plants (Selim et al., 2019).

Seawater has the potential to be used as an alternative source for irrigation of crop plants in order to address water scarcity and overpopulation. Furthermore, effective technologies, such as the use of magnetic fields, should be utilized to overcome the adverse effects of salinity on plant growth. However, to date, studies on using magnetic fields to reduce the negative effects of seawater are scarce. Therefore, the aim of the current study was to evaluate the impact of irrigation with magnetically treated seawater on germination, growth, and certain physiological, anatomical, and yield components of wheat plants.

## Materials and methods

### Experimental procedures

*In vitro* and pots experiments were conducted to investigate physiological and anatomical changes as well as the germination parameters of wheat cultivar, Sakha 93 irrigated with seawater of varying salinity levels and exposures to magnetic fields. The wheat grains were obtained from different resources, Ministry of Egyptian Agriculture and Land Reclamation, Giza, Egypt, and Field Crops Research Institute, Agricultural Research Center, Giza, Egypt. Sakha 93 is a stripe rust-resistant Egyptian cultivar that was introduced in 1990 (Shehab-El-Din et al., 1999).

Water was pumped *via* a magnetron device with conditions, a magnetic tube model U.T.I, one-inch diameter, production 4–6 m^3^ h^−1^ (Magnetic Technologies L.C.C., Dubai, United Arab Emirates).

The laboratory and pot trails were conducted as the following:

#### *In vitro* experiments

These experiments were carried out to study the germination parameters. Ten grains with five replicates were put in Petri plates containing moisturized Whatman No.1 filter paper.

#### *In vivo* experiments

Pot experiment was carried out in a greenhouse at the Faculty of Agriculture’s experimental farm in Shebin El-Kom, Egypt. This trial was carried out during two successive winter seasons (2018–19 and 2019–20), from ten November to 3 May; Supplementary Table S1 shows the chemical and physical parameters of the clay loam soil that was utilized in this study. Polyethylene pots (30 cm diameter × 30 cm depth) were packed with 8 kg of made soil. The experiment involved ten treatments with four replicates of each treatment. Fifteen grains of wheat without visible defect were sown in each pot. Plant samples (one plant sample from each replicate) were obtained on day 95 post-sowing at 7:00 AM for chemical and physiological assessments. The soil was amended with N, P, and K, based on the recommendation of the Ministry of Egyptian Agriculture and Land Reclamation, Giza, Egypt.

### Treatments

The experimental treatments were described as the following:

1. Irrigation using tap water with an EC of 0.40 dS m^−1^ (control) and various levels of diluted seawater (EC at 5.0, 7.5, 10.0, and 12.5 dS m^−1^) without magnetic treatment.
2. Irrigation with the same above-mentioned treatments but treated with exposure to a magnetic field by passing the water through a magnetron.

Irrigation was carried out with seawater with different levels of salinity (EC at 5.0, 7.5, 10.0, and 12.5 dS m^−1^) prepared from 100% synthetic seawater following the method of Lunin et al. (1961). The water was composed of NaCl, MgCl_2_.6H_2_O, CaSO_4_ and K_2_SO4 with the volume of 473:102:20:12 mEq.l^−1^, respectively. Irrigation was carried out manually, based on the field capability, by supplying the needed amount of prepared water.

### Studied traits

#### Germination parameters

Germination (%): the number of normal seedlings was counted 15 days after sowing (DAS), according to the following formula:


$$\begin{array}{c} \mathrm{Germination}\;\left(\%\right)=\mathrm{Number of germinated}\;\\ \;\;\mathrm{seeds/Total}\;\mathrm{number of seeds}\times 100\%. \end{array}$$

Mean germination time (MGT) was calculated according to the equation of Ellis and Roberts (1981), as follows:


MGT=∑Dn/∑n

where n is the number of seeds germinated on day D and D is the number of days counted from the beginning of germination.

Germination index (GI) or germination sprouting was determined according to the equation of Scott et al. (1984), as follows:


$$GI=\left(\sum T_{i}\;N_{i}\right)/\left(S\right)$$

where $T_{i}$ = Number of DAS; $N_{i}$ = Number of germinated seeds on day i; S, the total number of seeds.

Coefficient of velocity (CV) was estimated according to Scott et al. (1984) as follows:


CV=[(∑Ni)/(∑NiTi)]×100

where $N_{i}$, number of germinated seeds on day i; $T_{i}$, number of DAS.

Seedling growth characteristics, such fresh weight (FW; g 10 seedlings^−1^), and dry weight (DW; g 10 seedlings^−1^), radicle length (RL; cm), and plumule length (PL; cm), were also determined.

Vigor index (VI) was calculated using the formula according to Abdul-Baki and Anderson (1973):


VI=Germination(%)×Totalseedlinglength(cm)

#### Physiological and productive parameters

##### Growth measurements

Root length (RL; cm), plant height (PH; cm), number of leaves plant^−1^, shoot DW (g), root DW (g), and the entire plant DW (g), shoot/root ratio, leaf area (cm^2^ plant^−1^) were determined following the according to the procedure of disk and leaf area indices measured by Simane et al. (1993).

Flag leaf width, length and flag leaf area (cm^2^) were also measured according to the formula provided by Gardner et al. (2017):


$$\begin{array}{c} Flagleafarea\;\left(cm^{2}\right)=Flagleaflength\\ \times Flagleafwidth\times 0.75 \end{array}$$

##### Water relations parameters

The total water content (TWC) in leaves was estimated as previously described (Gossev, 1960).

The RWC and leaf water deficit (LWD) were calculated using the formula of Kalapos (1994):


RWC(%)=(Turgid weight−FW/Turgid weight−DW×100

LWD (%)=100−RWC

The osmotic pressure (atm) was obtained by using special tables following the method of Gossev (1960).

The transpiration rate (TR; mg cm^−2^ h^−1^) was estimated by the following formula (Kreeb, 1990):


$$\text{TR}=\left[\frac{\text{FW}-\text{Plant weight after 1hour}}{{\text{Plant area in cm}}^{2}}\right]\times 1000$$

The absorption of solute leakage crossways the cell membranes of tissues was evaluated at the 273 nm UV wavelength using a spectrophotometer (UV-2101/3101 PC; Shimadzu Corporation, Analytical Instruments Division, Kyoto, Japan) to determine the membrane integrity (MI; Leopold et al., 1981).

##### Chemical parameters

Photosynthetic pigments, such as chlorophyll (Chl) *a* and *b* and carotenoids (Car), were determined using a spectrophotometer (Fadeel, 1962).

UV-absorbing substances (UVAS) were determined in the leaves according to Lichtenthaler (1987), as follows: leaf samples (1.0 g) were boiled in 100 ml of water. After discarding the leaves, the residue was resolved in 10 ml of methanol. The absorbance of extracts was measured at 300 nm using a UV spectrophotometer and the concentration of UVAS was expressed as E300 nm g FW^−1^.

Total soluble sugars (TSS; mg g DW^−1^) and total free amino acids (FAA) in the fine dry leaf powder were separated with chloroform: methanol (3:7, v/v) on ice for 30 min according to the method of Lohaus et al. (2000).

To determine the content of Pro (μmol g FW^−1^), 3% sulphosalicylic acid was recovered from fresh wheat leaves (Bates et al., 1973).

Phenoloxidase (EC 1.14.18.1) and peroxidase (EC 1.11.1.7) activities were determined according to the previous methods (Kar and Mishra, 1976) as the following: Ten grams of fresh leaves from each treatment was separately homogenized in liquid nitrogen and ground with 10 ml phosphate buffer (pH 7.0). Extracts were then centrifuged at 12,000 x *g*, for 15 min at less than 4°C. The terminal volume of the supernatant was adjusted to 10 ml by supplying distilled water and this final solution was considered as the source of the enzymes. The color intensity was measured at 430 nm, and enzyme activity was calculated as a modification in optical density g FW^−1^ h^−1^.

For the mineral elements, 0.2 g of dried powdered leaves was digested in concentrated H_2_SO_4_ and H_2_O_2_ (5:1) for chemical analyses of nitrogen (N), phosphorus (P), potassium (K), iron (Fe), and chloride (Cl; AOAC, 1995); while sodium (Na) was assessed using a flame photometer (Model: 400 FP).

##### Concentration of phytohormones

The endogenous phytohormone levels in the leaves of the wheat plants were determined 65 DAS and hormone concentrations were estimated using high-pressure liquid chromatography (HPLC; Crozier and Moritz, 1999).

##### Components of yield at harvest

The yield components, spike length (SpL; cm), spike weight (SpW; g plant^−1^), grains number.spike^−1^ (GNSp), grains weight (g plant^−1^), 100 grains weight (GI; g), and straw yield (g plant^−1^) were also estimated.

#### Anatomical characteristics

Flag leaves and the fourth upper internode of stem samples were collected at 85 DAS during the second growing season (2019/2020). In FAA, specimens were fixed in fixation solution (5 ml glacial acetic acid, 10 ml formaldehyde, and 85 ml of ethanol 70%) for 2 days. The fixed specimens were cleaned in 50% ethanol, dehydrated in a series of butanol concentrations, embedded in melted paraffin wax at 56°C, segmented to a 20 μm thickness, then stained in safranin-light green, and destained in xylene and mounted in Canada balsam based method (Nassar and El-Sahhar, 1998). Pieces were photomicrographic and examined using Olympus BH-2 (Olympus Optical Co. Ltd, Tokyo, Japan) light microscope equipped with a digital camera and software (Jenoptik ProgRes Camera, C12plus, Frankfurt, Germany).

### Statistical analysis

The data means were analyzed by analysis of variance (ANOVA) using SAS software, v 9.2 (SAS, 2014). The ANOVA examined the differences among the means of interaction among the two main factors (magnetic water treatments at two levels; magnetic and non-magnetic water) and seawater levels (five levels: control, 5.0, 7.5, 10.0, and 12.5 dS m^−1^). The significant differences between means were adopted *via* Duncan’s New Multiple Range Test at 5% significance degree.

## Results

### Germination and seedling characteristics

Germination and seedling parameters of Sakha 93 wheat seeds irrigated with different seawater levels and exposed to magnetic treatment are shown in Table 1. As salinity stress increased, the germination and seedling parameters decreased significantly (*p* < 0.05). Examining seawater at 12.5 dS m^−1^, the largest drop in germination % and DW of seedlings was observed at 15% and 20%, respectively, when compared to their controls.

**Table 1:** Effect of seawater stress levels, magnetic treatments on germination, and seedling characters of wheat grains.

Magnetic treatment	(dS m^−1^)	Germination (%)	MGT (days)	GI	CV	(FW g 10 seedlings^−1^)	(DW; g 10 seedlings^−1^)	RL (cm)	PL (cm)	VI
No magnetic	Control	90.00^bc^	4.38^a^	16.84^ab^	54.11^c^	2.82^ab^	0.284^a^	12.91^bc^	14.19^ab^	24.42^c^
	5	83.33^de^	4.64^a^	12.44^d^	34.72^e^	2.78^ab^	0.248^a^	11.35^cd^	14.64^a^	21.50^d^
	7.5	80.00^bc^	4.56^a^	12.15^d^	32.44^ef^	2.55^ab^	0.283^a^	9.57^de^	12.92^abc^	17.99^ef^
	10	80.00^ef^	4.64^a^	12.08^d^	33.11^ef^	2.59^ab^	0.281^a^	7.83^ef^	13.62^abc^	17.15^f^
	12.5	76.67^f^	4.59^a^	11.93^d^	30.72^f^	1.78^b^	0.226^a^	6.89^f^	11.71^c^	14.30^g^
Magnetic water	Control	96.67^a^	4.29^a^	19.03^a^	65.72^a^	3.07^a^	0.304^a^	15.43^a^	14.30^ab^	28.63^a^
	5	90.00^bc^	4.26^a^	18.24^a^	59.00^b^	2.84^ab^	0.287^a^	14.67^ab^	14.88^a^	26.60^b^
	7.5	93.33^ab^	4.31^a^	18.11^a^	59.72^b^	2.88^a^	0.286^a^	11.03^cd^	14.20^ab^	23.52^c^
	10	83.33^de^	4.42^a^	15.22^bc^	45.03^d^	2.68^ab^	0.288^a^	9.10d^ef^	13.82^abc^	19.07^e^
	12.5	86.67^cd^	4.47^a^	14.57^c^	43.79^d^	2.08^ab^	0.238^a^	7.49^ef^	11.98^bc^	16.88^f^

Values followed by different letters within a column are significantly different (*p* < 0.05) according to Duncan’s new multiple range test. MGT, mean germination time; GI, germination index; CV, coefficient of velocity; FW, fresh weight; DW, dry weight; RL, radicle length; PL, plumule length; and VI, vigor index.

### Growth characteristics

Growth characteristics of wheat plants as affected by different levels of magnetically treated seawater are shown in Table 2. During both seasons, the growth parameters reduced significantly (*p* < 0.05) as seawater level increased. With a seawater level of 12.5 dS m^−1^, the DM deficit in whole plants was 33% and 49% in the first and second seasons, respectively, compared to the control. With a seawater level of 7.5 dS m^−1^, significant increases of 36% and 69% in leaf area index and flag leaf area of wheat plants were obtained utilizing magnetically treated seawater compared to the non-magnetic treatment in the first growing season. In this respect, the results of both seasons are similar.

**Table 2:** Effect of seawater stress levels, magnetic treatment on some vegetative growth characters of wheat plants during the first and second growing seasons.

Characteristic magnetic treatment		RL (cm)	PH (cm)	Number of leaves plant^−1^	DW (g plant^−1^)			S/R ratio	LA (cm^2^ plant^−1^)	LAI	FLL (cm)	FLW (cm)	FLFW (g plant^−1^)	FLDW (g plant^−1^)	FLA (cm^2^)
Treatment	dS m^−1^				R	S	W								
First season															
No magnetic	Control	4.83^b^	49.00^a^	7.00^ab^	0.020^cd^	0.472^de^	0.492^de^	23.18^ab^	153.17^ef^	1.219^de^	12.67^cd^	0.900^ef^	0.253^abc^	0.080^cd^	8.30^d^
	5	5.67^b^	48.17^ab^	6.00^bc^	0.020^cd^	0.435^de^	0.455^de^	21.75^abc^	148.96^f^	1.186^def^	12.33^d^	1.033^cd^	0.207^bcd^	0.070^cd^	9.58^c^
	7.5	5.00^b^	43.50^c^	6.00^bc^	0.017^d^	0.377^ef^	0.393^ef^	22.89^ab^	141.95^g^	1.130^efg^	12.33^d^	0.833^f^	0.180^cd^	0.078^cd^	7.30^d^
	10	3.83^c^	43.00^c^	5.67^c^	0.014^d^	0.337^f^	0.351^f^	24.06^ab^	137.10^gh^	1.092^fg^	11.67^d^	0.833^f^	0.217^bc^	0.063^d^	7.93^d^
	12.5	3.67^c^	32.67^e^	5.33^c^	0.015^d^	0.317^f^	0.331^f^	22.50^a^	134.91^h^	1.074^g^	8.67^e^	0.700^g^	0.105^d^	0.055^e^	4.60^e^
Magnetic water	Control	6.83^a^	49.17^a^	7.33^a^	0.047^a^	1.040^a^	1.087^a^	22.03^abc^	220.83^a^	1.758^a^	15.33^a^	1.167^ab^	0.303^ab^	0.142^a^	12.30^b^
	5	7.00^a^	48.33^ab^	7.33^a^	0.040^ab^	0.868^b^	0.908^b^	22.51^abc^	200.48^b^	1.596^b^	15.67^a^	1.233^a^	0.338^a^	0.152^a^	14.38^a^
	7.5	5.83^b^	45.67^ab^	7.67^a^	0.037^b^	0.800^b^	0.836^b^	22.49^abc^	192.30^c^	1.531^b^	14.00^b^	1.167^ab^	0.267^abc^	0.127^b^	12.35^b^
	10	5.17^b^	44.00^bc^	6.00^bc^	0.029^c^	0.610^c^	0.639^c^	21.33^bc^	169.87^d^	1.352^c^	14.00^b^	0.967^de^	0.207^bcd^	0.078^c^	11.08^b^
	12.5	5.33^b^	38.67^d^	6.00^bc^	0.025^cd^	0.487^d^	0.511^d^	20.19^c^	155.36^e^	1.237^d^	13.67^bc^	1.100^bc^	0.203^bcd^	0.072^cd^	11.35^b^
Second season															
No magnetic	Control	4.50^cd^	48.83^a^	7.33^ab^	0.020^c^	0.515^e^	0.535^f^	25.35^a^	158.09^f^	1.259^f^	14.33^ab^	0.933^bc^	0.253^abc^	0.080^b^	9.98^c^
	5	5.00^bc^d	47.33^a^	6.00^c^	0.019^c^	0.472^e^	0.491^f^	24.38^ab^	153.05^g^	1.219^f^	12.00^def^	0.933^bc^	0.207^bcd^	0.077^bc^	8.43^de^
	7.5	5.00^bcd^	43.67^b^	6.33^bc^	0.018^c^	0.437^e^	0.455^f^	24.72^ab^	148.92^h^	1.186^f^	11.67^ef^	0.833^cd^	0.180^cd^	0.070^cd^	7.30^ef^
	10	4.00^d^	43.00^b^	6.33^bc^	0.015^c^	0.315^f^	0.330^g^	22.53^cd^	134.76^i^	1.073^g^	11.00^f^	0.800^d^	0.163^cd^	0.067^cd^	6.63^cd^
	12.5	4.00^d^	34.00^d^	5.33^c^	0.013^c^	0.262^f^	0.274^g^	20.88^de^	128.43^j^	1.023^g^	8.67^g^	0.700^e^	0.105^d^	0.047^e^	4.60^cd^
Magnetic water	Control	6.67^a^	49.33^a^	7.67^a^	0.043^b^	1.093^a^	1.136^a^	25.51^a^	226.39^a^	1.803^a^	15.00^a^	1.167^a^	0.320^a^	0.113^a^	13.18^ab^
	5	7.00^a^	49.00^a^	7.33^ab^	0.065^a^	0.960^b^	1.024^b^	25.67^a^	213.65^b^	1.701^b^	15.00^a^	1.233^a^	0.319^ab^	0.112^a^	13.78^a^
	7.5	5.33^bc^	46.33^ab^	8.00^a^	0.045^b^	0.875^b^	0.920^c^	23.12^bc^	201.77^c^	1.606^c^	13.67^bc^	1.167^a^	0.267^abc^	0.098^a^	12.05^b^
	10	5.33^bc^	43.33^b^	6.00^c^	0.037^b^	0.727^c^	0.764^d^	20.49^e^	184.04^d^	1.465^d^	13.00^cd^	0.967^b^	0.207^bcd^	0.065^d^	9.45^cd^
	12.5	6.00^ab^	39.00^c^	6.00^c^	0.034^b^	0.617^d^	0.651^e^	18.13^f^	171.24^e^	1.363^e^	12.67^cde^	0.933^bc^	0.203^cd^	0.063^d^	8.85^cd^

Values followed by different letters within a column are significantly different (*p* < 0.05) according to Duncan’s new multiple range test. RL, root length; PH, plant height; DW, dry weight; R, root; S, shoot; W, whole; LA, leaf area; LAI, leaf area index; FLL, flag leaf length; FLW, flag leaf width; FLFW, flag leaf fresh weight; FLDW, flag leaf dry weight; and FLA, flag leaf area.

### Water relations

During both seasons, increased seawater levels resulted in a significant decrease in water relations values (Table 3). The RWC (%) shortfall was 12% and 33% in the first and second seasons, respectively, as compared to the control, at a seawater level of 12.5 dS m^−1^.

**Table 3:** Effect of seawater stress levels, magnetic treatment on water relation in leaves of wheat plants during the first and second growing seasons.

Characters treatments		TWC (%)	RWC (%)	LWD (%)	Osmotic pressure (atm)	TR (mg cm^−2^ h^−1^)	MI (%)
Magnetic treatments	dS m^−1^						
First season							
No magnetic	Control	71.56^bc^	49.78^c^	50.22^e^	7.71^a^	1.139^a^	22.06^g^
	5	71.41^bc^	35.72^f^	64.28^b^	7.89^a^	1.044^a^	26.19^ef^
	7.5	68.53^cd^	44.66^d^	55.34^d^	8.00^a^	0.893^b^	27.90^b^
	10	69.11^cd^	33.89^fg^	66.11^ab^	8.15^a^	0.870^b^	39.58^b^
	12.5	66.51^d^	31.43^g^	68.57^a^	8.26^a^	0.834^bc^	41.30^b^
Magnetic water	Control	76.44^a^	54.55^ab^	45.45^fg^	7.89^a^	0.903^b^	25.43^f^
	5	72.88^b^	56.64^a^	43.36^g^	8.11^a^	0.879^b^	30.34^d^
	7.5	69.06^d^	52.23^bc^	47.77^ef^	8.33^a^	0.858^b^	35.16^c^
	10	69.98^cd^	34.72^f^	65.28^b^	8.44^a^	0.752^c^	40.00^b^
	12.5	68.50^cd^	39.03^e^	60.97^c^	8.55^a^	0.750^c^	43.72^a^
Second season							
No magnetic	Control	79.37^a^	59.46^c^	40.54^e^	7.60^a^	1.122^a^	15.54^g^
	5	77.38^ab^	41.27^e^	58.73^c^	7.64^a^	1.054^ab^	23.12^e^
	7.5	73.08^c^	59.42^c^	40.59^e^	7.86^a^	0.932^cd^	26.89^d^
	10	68.90^d^	35.51^f^	64.49^b^	7.89^a^	0.882^d^	34.01^c^
	12.5	60.00^e^	21.34^g^	78.66^a^	8.07^a^	0.782^e^	34.78^c^
Magnetic water	Control	81.42^a^	59.86^c^	40.15^e^	6.49^b^	0.991^bc^	20.90^f^
	5	78.53^a^	65.83^b^	34.17^f^	7.89^a^	0.909^cd^	32.84^c^
	7.5	78.01^a^	89.40^a^	30.60^g^	8.04^a^	0.890^cd^	45.48^a^
	10	73.70^bc^	43.17^e^	56.83^c^	8.11^a^	0.743^ef^	44.54^a^
	12.5	71.29^cd^	46.76^d^	53.24^d^	8.18^a^	0.646^f^	39.26^b^

Values followed by different letters within a column are significantly different (*p* < 0.05) according to Duncan’s new multiple range test. TWC, total water content; RWC, relative water content; LWD, leaf water deficit; TR, transpiration rate; and MI, membrane integrity.

### Chemical characteristics

#### Photosynthetic pigments

When seawater stress increased, the photosynthetic pigment content in the wheat plants reduced dramatically compared to the control (Supplementary Table S2). With a seawater level of 12.5 dS m^−1^, the decrease in total chlorophyll (*a* + *b*) was 31% and 27% lower in the first and second seasons, respectively, compared to the control. These changes boost photosynthetic efficiency. Additionally, magnetic treatments increased the synthesis of ultraviolet absorbing compounds in the leaves, making them more resistant to harmful UV-B radiation.

#### TSS, FAA, and Pro concentrations

Table 4 shows that the entire soluble sugar concentration in wheat plant leaves decreased by approximately 36% when non-magnetic seawater level was increased to 12.5 dS m^−1^ compared to the control during the second season. In the second season, FAA and Pro were enhanced in wheat plant leaves by approximately 11% and 250%, respectively, under a stress level of 12.5 dS m^−1^, compared to the control. The same trend in grains was observed (Table 4). In the second season, however, TSS concentration in leaves treated with magnetic water increased by approximately 45% and 65% under seawater levels of 10 and 12.5 dS m^−1^, respectively, when compared to plants treated with non-magnetically treated water. In the second season, as compared to untreated plants, the application of magnetically treated water elevated whole FAA and Pro concentration in leaves by approximately 10 and 80%, respectively, at a seawater level of 12.5 dS m^−1^.

**Table 4:** Effect of seawater stress levels, magnetic treatment on some chemical constituents in shoot and grains of wheat plants during the second growing season.

Characters treatments		Shoot					Grains		
		TSS	FAA	Pro	Enzymes activity		TSS	FAA	Pro
					Phenoloxidase	Peroxidase			
Magnetic treatments	dS m^−1^	mg g DW^−1^		μmol g FW^−1^	OD g FW^−1^		mg g DW^−1^	μmol g FW^−1^	
No magnetic	Control	242.19^f^	97.20^d^	1.45^f^	0.65^cde^	0.45^f^	369.12^c^	88.78^f^	0.36^h^
	5	234.38^g^	97.20^d^	2.91^de^	0.74^c^	0.68^e^	334.38^e^	102.20^e^	1.02^f^
	7.5	203.13^h^	113.40^bc^	3.34^de^	0.61^de^	0.54^f^	318.11^f^	103.40^e^	1.23^e^
	10	187.5^i^	102.60^d^	3.78^d^	0.59^de^	0.50^f^	251.10^i^	112.60^d^	1.45^d^
	12.5	156.25^j^	108.00^c^	5.08^c^	0.58^e^	0.90^d^	236.29^j^	110.09^d^	2.25^c^
Magnetic water	Control	390.63^a^	113.40^bc^	2.62^e^	0.68^cd^	0.95^d^	412.73^a^	153.40^a^	0.80^g^
	5	312.50^b^	113.40^bc^	3.63^d^	1.01^a^	1.71^a^	400.50^b^	141.40^c^	1.45^d^
	7.5	281.25^c^	118.80^ab^	5.23^c^	0.86^b^	1.26^b^	351.25^d^	146.50^b^	2.18^c^
	10	272.42^d^	124.20^a^	7.70^b^	0.72^c^	0.99^d^	293.49^h^	138.40^c^	3.12^b^
	12.5	256.94^e^	118.80^ab^	9.15^a^	0.72^c^	1.13^c^	302.81^g^	148.80^b^	3.63^a^

Values followed by different letters within a column are significantly different (*p* < 0.05) according to Duncan’s new multiple range test. TSS, total soluble sugars; FAA, free amino acids; Pro, proline; DW, dry weight; and FW, fresh weight.

#### Phenoloxidase and peroxidase enzymes activity

The activity of phenoloxidase enzymes decreased as seawater levels increased, whereas the activity of peroxidase enzymes increased significantly in wheat plant leaves (Table 4). With a seawater level of 10 dS m^−1^, the reduction in phenoloxidase activity was approximately 9%, and the increase in peroxidase activity was approximately 11%, respectively, compared to the control.

#### Mineral concentrations

Table 5 shows that as seawater levels rose, macro and micro minerals in wheat plants and grains declined with the exception of Na and Cl, which increased. With a 12.5 dS m^−1^ seawater level, the concentrations of the elements N, P, K, and Fe in the wheat plant shoots declined by approximately 19%, 27%, 13%, and 69%, respectively, compared to the control. When examining all seawater levels, the magnetic treatment caused significant increases in concentration of all the minerals, except Na and Cl, in all areas of wheat plants. In comparison to the non-magnetic treatment, the N, P, and K concentrations in grains increased by roughly 50%, 24% and 25%, respectively, while the Na and Cl concentrations in the shoot decreased by approximately 20% and 25%, at a seawater level of 12.5 dS m^−1^.

**Table 5:** Effect of seawater stress levels, magnetic treatment on the concentrations of some elements in different organs of wheat plants during the second growing season.

Characters treatments		Root						Shoot						Grains				
		N	P	K	Cl	Na	Fe	N	P	K	Cl	Na	Fe	N	P	K	Cl	Na
Magnetic treatments	dS m^−1^	(%)	(%)	(%)	(ppm)	(ppm)	(ppm)	(%)	(%)	(%)	(ppm)	(ppm)	(ppm)	(%)	(%)	(%)	(ppm)	(ppm)
No magnetic	Control	2.312^b^	0.163^a^	2.653^abcd^	3.053^f^	1.418^g^	310.20^b^	2.604^ab^	0.250^ab^	3.511^b^	2.272^de^	0.409^bc^	351.00^c^	2.520^e^	0.483^b^	0.729^bc^	0.639^cd^	0.172^a^
	5	2.100^b^	0.154^a^	2.229^bcd^	3.408^e^	1.518^f^	284.80^d^	2.436^b^	0.218^ab^	3.301^cd^	2.769^cde^	0.377^cd^	321.00^e^	2.520^e^	0.455^bc^	0.733^bc^	0.675^c^	0.183^a^
	7.5	1.977^b^	0.150^a^	1.786^cd^	3.692^d^	2.130^c^	230.80^g^	2.294^b^	0.213^ab^	3.200^d^	3.053^bcd^	0.583^a^	274.20^g^	2.272^f^	0.429^bc^	0.694^c^	0.710^c^	0.192^a^
	10	2.040^b^	0.148^a^	1.707^d^	4.402^b^	2.283^b^	185.34^i^	2.152^b^	0.199^ab^	3.057^e^	3.692^bc^	0.583^a^	207.20^h^	2.040^g^	0.378^c^	0.686^c^	0.852^b^	0.197^a^
	12.5	1.004^c^	0.141^a^	1.662^d^	5.183^a^	2.507^a^	170.00^j^	2.100^b^	0.182^b^	3.043^e^	5.112^a^	0.583^a^	108.40^i^	1.904^h^	0.356^c^	0.562^d^	0.994^a^	0.200^a^
Magnetic water	Control	3.876^a^	0.221^a^	3.391^a^	2.272^i^	1.076^h^	365.22^a^	3.528^a^	0.304^a^	3.692^a^	1.988^e^	0.216^e^	376.80^a^	3.024^b^	0.611^a^	0.822^a^	0.497^e^	0.115^a^
	5	2.407^b^	0.193^a^	3.043^ab^	2.485^h^	1.352^g^	291.02^c^	2.688^ab^	0.228^ab^	3.482^b^	2.272^de^	0.309^d^	361.00^b^	3.192^a^	0.497^b^	0.793^ab^	0.568^de^	0.133^a^
	7.5	2.342^b^	0.184^a^	2.818^abc^	2.840^g^	1.536^f^	266.82^e^	2.468^b^	0.241^ab^	3.300^c^	2.698^cde^	0.425^bc^	342.20^d^	2.772^d^	0.606^a^	0.772^abc^	0.639^cd^	0.138^a^
	10	2.309^b^	0.180^a^	2.385^abcd^	3.621^d^	1.783^e^	239.02^f^	2.352^b^	0.233^ab^	3.271^cd^	3.053^bcd^	0.471^b^	321.60^e^	2.940^bc^	0.530^ab^	0.762^abc^	0.710^c^	0.152^a^
	12.5	2.105^b^	0.188^a^	2.026^bcd^	4.189^c^	1.907^d^	211.22^h^	2.290^b^	0.221^ab^	3.229^cd^	3.834^b^	0.469^b^	300.20^f^	2.856^cd^	0.442^bc^	0.702^bc^	0.923^ab^	0.157^a^

Values followed by different letters within a column are significantly different (*p* < 0.05) according to Duncan’s new multiple range test.

#### Concentration of phytohormones

The concentrations of indole-3-acetic acid (IAA), cytokinins (CKs) and gibberellic acid (GA_3_) decreased by approximately 28%, 19%, and 14%, respectively, with increasing seawater levels; while the concentration of abscisic acid (ABA) increased by about 90% at a 10 dS m^−1^ seawater level compared to the control (Table 6). The influence of the magnetic field resulted in an increase in growth phytohormone levels and had positive effects on the mineral uptake, Chl synthesis and plant growth.

**Table 6:** Effect of seawater stress levels, magnetic treatment on concentration of plant phytohormones in the shoot of wheat plants during the second growing season.

Characters treatments		IAA	CK	GA_3_	ABA	Activators/inhibitors ratio
Magnetic treatments	dS m^−1^	(μg 100 g FW^−1^)		(mg 100 g FW^−1^)		
No magnetic	Control	897.38^a^	338.7^b^	29.68^c^	2.55^b^	12.124^c^
	10	643.39^b^	272.99^b^	25.53^c^	4.85^a^	5.453^d^
Magnetic water	Control	958.2^a^	443.56^a^	64.78^a^	1.20^c^	55.151^a^
	10	683.31^b^	442.59^a^	54.51^b^	1.50^bc^	37.091^b^

Values followed by different letters within a column are significantly different (*p* < 0.05) according to Duncan’s new multiple range test. IAA, indole acetic acid; CKs, cytokinins; GA_3_, gibberellic acid; ABA, abscisic acid; and FW, fresh weight.

### Yield attributes

Table 7 shows that all yield features of wheat plants [SpL (cm), SpW (g plant^−1^), GNSp, grain weight (g plant^−1^), weight of 100 grains (g), and straw yield (g plant^−1^)] dropped significantly with increasing seawater levels during both seasons. In the first and second seasons, the reduction in grain weight was 91% and 68%, respectively, at the elevated seawater of 12.5 dS m^−1^ in contrast to the control. The irrigation with magnetic water; however, increased the previous yield attributes by roughly 16%, 30%, 58%, 688%, 37%, and 11%, respectively, at a seawater stress level of 10 dS m^−1^ in the first season, compared to controls. The second season followed the same pattern.

**Table 7:** Effect of seawater stress levels, magnetic treatment, and their interactions on yield attributes of wheat plants during the first and second growing seasons.

Characters treatments		SpL (cm)	SpW (g plant^−1^)	GNSp	Grains weight (g plant^−1^)	GI (g)	Straw weight (g plant^−1^)
Magnetic treatments	dS m^−1^						
First season							
No magnetic	Control	6.40^ab^	0.320^cd^	8.75^cd^	0.352^c^	2.500^b^	0.409^b^
	5	5.80^ab^	0.290^d^	6.25^ef^	0.180^g^	1.500^c^	0.335^d^
	7.5	5.80^ab^	0.270^d^	5.00^f^	0.053^h^	1.100^d^	0.336^d^
	10	5.70^ab^	0.270^d^	4.75^f^	0.040^i^	0.750^f^	0.323^d^
	12.5	4.00^c^	0.130^e^	3.00^g^	0.031^j^	0.375^g^	0.271^f^
Magnetic water	Control	7.00^a^	0.460^a^	12.25^a^	0.560^a^	3.500^a^	0.471^a^
	5	6.80^ab^	0.430^ab^	10.75^ab^	0.398^b^	2.500^b^	0.365^c^
	7.5	6.90^ab^	0.380^abc^	9.75^bc^	0.340^d^	1.500^c^	0.361^c^
	10	6.60^ab^	0.350^bcd^	7.50^ed^	0.315^e^	1.025^d^	0.357^c^
	12.5	5.60^b^	0.290^d^	5.50^f^	0.300^f^	0.925^e^	0.307^e^
Second season							
No magnetic	Control	10.50^bc^	0.384^cd^	13.40^c^	0.285^de^	2.132^bc^	1.307^cd^
	5	10.70^bc^	0.341^ef^	10.80^d^	0.221^ef^	2.070^bc^	1.016^ef^
	7.5	10.30^c^	0.381^fg^	11.60^d^	0.201^f^	1.975^c^	0.819^fg^
	10	9.10^d^	0.323^h^	8.40^e^	0.151^fg^	1.962^c^	0.444^h^
	12.5	9.00^d^	0.168^g^	4.80^g^	0.090^g^	1.923^c^	0.714^g^
Magnetic water	Control	13.60^a^	1.207^a^	27.00^a^	1.013^a^	3.018^a^	2.900^a^
	5	11.50^b^	0.710^b^	19.60^b^	0.683^b^	2.856^a^	2.061^b^
	7.5	10.70^bc^	0.772^c^	18.80^b^	0.518^c^	2.792^a^	1.495^c^
	10	10.50^bc^	0.579^de^	13.20^c^	0.366^d^	2.713^ab^	1.100^de^
	12.5	10.20^c^	0.256^de^	6.40^f^	0.164^fg^	2.531^abc^	1.140^de^

Values followed by different letters within a column are significantly different (*p* < 0.05) according to Duncan’s new multiple range test. SpL, spike length; SpW, spike weight; GNSp, grains number spike^−1^; and GW, 100 grains weight.

### Anatomical structure of leaf blade and stem

**Figure 1:**
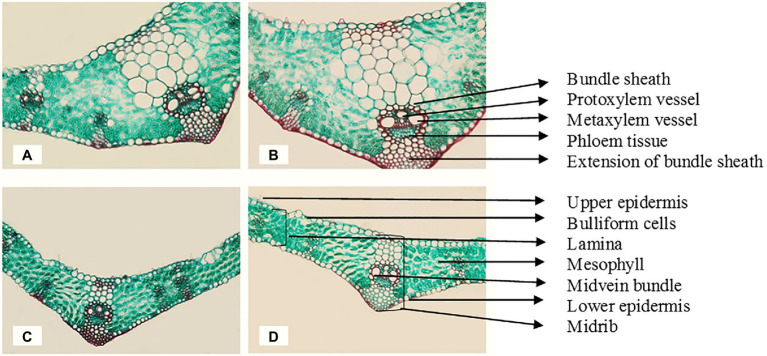
Transverse cross sections of the marginal part of the flag leaf blade at the main stem of wheat cv. Sakha 93 at the heading stage (X 300). **(A)** Control (from plants irrigated with normal tap water); **(B)** magnetic treatment (from plants irrigated with magnetized water); **(C)** seawater treatment (from plants irrigated with seawater at 10 dS m^−1^ level); **(D)** magnetic seawater treatment (from plants irrigated with magnetized seawater at 10 dS m^−1^ level).

**Figure 2:**
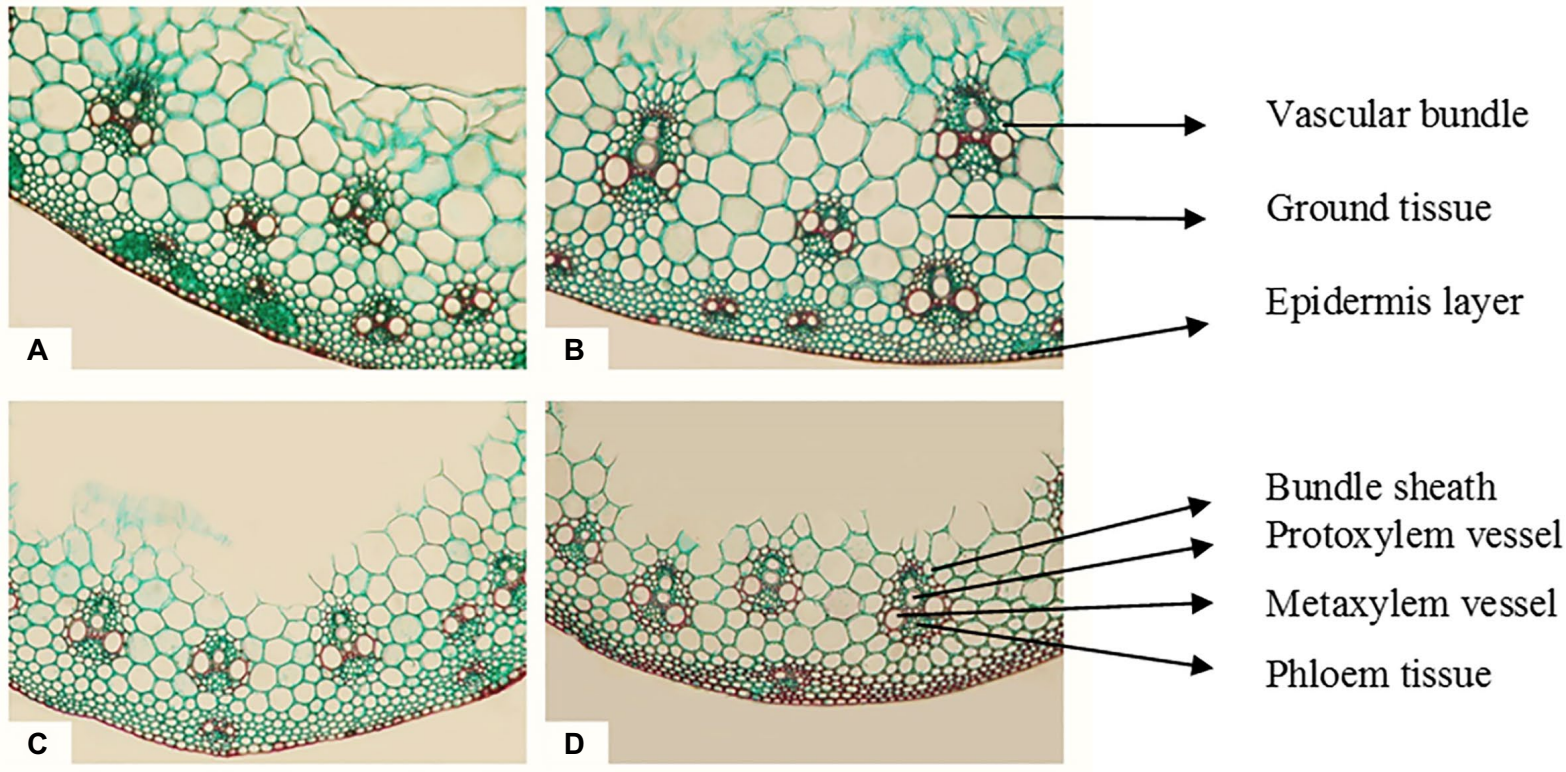
Transverse cross sections of the main stem of wheat cv. Sakha 93 at the heading stage (X 300). **(A)** Control (from plants irrigated with normal tap water); **(B)** magnetic treatment (from plants irrigated with magnetized water); **(C)** seawater treatment (from plants irrigated with seawater at 10 dS m^−1^ level); **(D)** magnetic seawater treatment (from plants irrigated with magnetized seawater at 10 dS m^−1^ level).

The magnetic technology utilized in this study had a strong positive effect and reduced the negative impacts of seawater irrigation on all the previously described characteristics of Sakha 93 wheat plants. In addition, the internal composition of the flag leaf and stem of wheat plants developed underneath seawater stress subjected to a magnetic field should be studied. In comparison with the control, wheat plants treated with magnetic technology and seawater at 10 dS m^−1^ exhibited histological features of flag leaf formation and stems (Table 8; Figures 1, 2). In the second season, at a seawater level of 10 dS m^−1^ the deficits in lamina thickness (μm), midvein bundle dimensions (μm), stem diameter (mm), and vascular bundle thickness (μm) were approximately 29%, 11%, 40%, and 12%, respectively, as contrasted to the control.

**Table 8:** Effect of seawater stress levels, magnetic treatment on some anatomical parameters in flag leaf and stem of wheat plants during the second growing season.

Treatments characters		0.40 dS m^−1^ (Control)		10 dS m^−1^					
		No magnetic	Magnetic water	±% to control	No magnetic	±% to control	Magnetic water	±% to control	±% to (10 dS m^−1^)
Flag leaf	Lamina thickness (μm)	170^ab^	200^a^	+18	120^c^	−29	140^bc^	−18	+17
	Midrib thickness (μm)	340^b^	420^a^	+24	200^d^	−41	280^c^	−18	+40
	Midvein bundle dimensions (μm)	120.7^b^	154.5^a^	+28	107.9^b^	−11	116.2^b^	−4	+8
	Metaxylem vessel diameter (μm)	29.6^b^	47.4^a^	+60	16.6^c^	−44	33.2^b^	+12	+100
Stem	Stem diameter (mm)	2.80^ab^	3.22^a^	+15	1.68^b^	−39	1.96^b^	−30	+15
	Stem cavity diameter (mm)	149^a^	2.48^b^	+66	0.94^b^	−40	1.24^b^	−17	+38
	Number of vascular bundle cross section^−1^	56^ab^	69^a^	+23	38^b^	−32	49^b^	−13	+29
	Vascular bundle thickness (μm)	100.2^c^	175.8^a^	+75	88.0^c^	−12	122.2^b^	+22	+39
	Metaxylem vessel diameter (μm)	23.5^c^	45.6^a^	+94	18.5^c^	−21	32.0^b^	+36	+73

Values followed by different letters within a column are significantly different (*p* < 0.05) according to Duncan’s new multiple range test.

Magnetic water treatment raised the lamina and midrib thickness (μm) of the flag leaf by approximately 17% and 40%, respectively, at the 10 dS m^−1^ level when compared to the control. Magnetic seawater of 10 dS m^−1^ increased the midvein bundle and metaxylem vessel diameter (μm) by 8% and 100%, respectively, as compared to the control. In comparison to untreated plants, the application of magnetically treated water increased stem diameter (mm) and stem cavity diameter (mm) by approximately 17% and 32%, respectively, at seawater levels of 10 dS m^−1^. The magnetic seawater at level 10 dS m^−1^ increased the number of vascular bundle cross sections, thickness, and metaxylem vessel diameter (μm) of the stem by 29%, 39%, and 73%, respectively compared to the non-magnetic treatment.

### Path analysis

**Figure 3:**
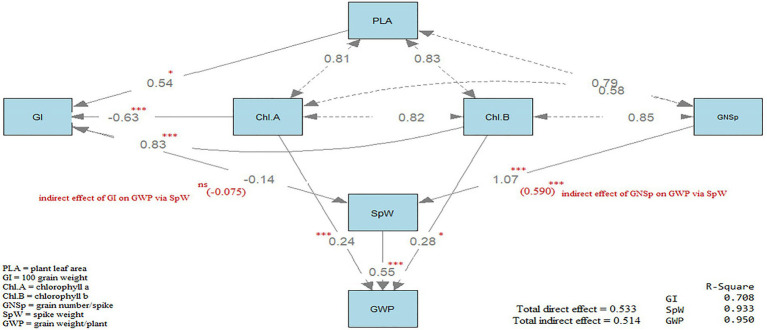
Path diagram showing the direct and indirect effects on wheat grain yield.

Path analysis (Tabachnick and Fidell, 1996) was accomplished using R statistical software version 4.1.0, 2021 using the package (lavaan) and the function (sem), which is the abbreviation of structural equation modeling. Path diagram was generated by using the function (semPaths) in the same package. Path analysis results are shown on the path diagrams in Figure 3. Three types of arrows are shown on the path diagram. The first type is representing the path; it is a single-headed arrow and is representing the causal relationships between the two variables, independent and dependent, the independent locates at the tail of the arrow while the dependent variable locates at the head of the arrow. The second type is representing the covariance between two variables; it is a double-headed arrow. The third type is representing the variance between two variables, it is a double-headed; however, the arrow is pointed at the same variable. Path diagram (Figure 3) included three direct effects and two indirect effects.

Concerning the direct effects, the first is the direct effect of plant leaf area (PLA), Chl *a* and *b* on GI where R^2^ value was 0.708. The second, is the direct effect of GI and GNSp where R^2^ value was 0.933.

The third, is the direct effect of SpW, Chl *a* and Chl *b* on grain weight plant^−1^ (GWP) where *R*^2^ value was 0.950. On the other hand, the indirect effects were two effects, the first is the indirect effect of plant leaf area (PLA), Chl *a* and Chl *b* on SpW, *via* GI. The second is the indirect effect of GI and GNSp on GWP *via* SpW. All the direct effects were significant (*p* < 0.05) except for GI on SpW. All the indirect effects were insignificant except for the indirect effect of GNSp on GWP *via* SpW. The total direct effects (0.533) were almost equal to the total indirect effects (0.514). In conclusion, GWP was affected directly and indirectly through interrelationships among yield components.

**Figure 4:**
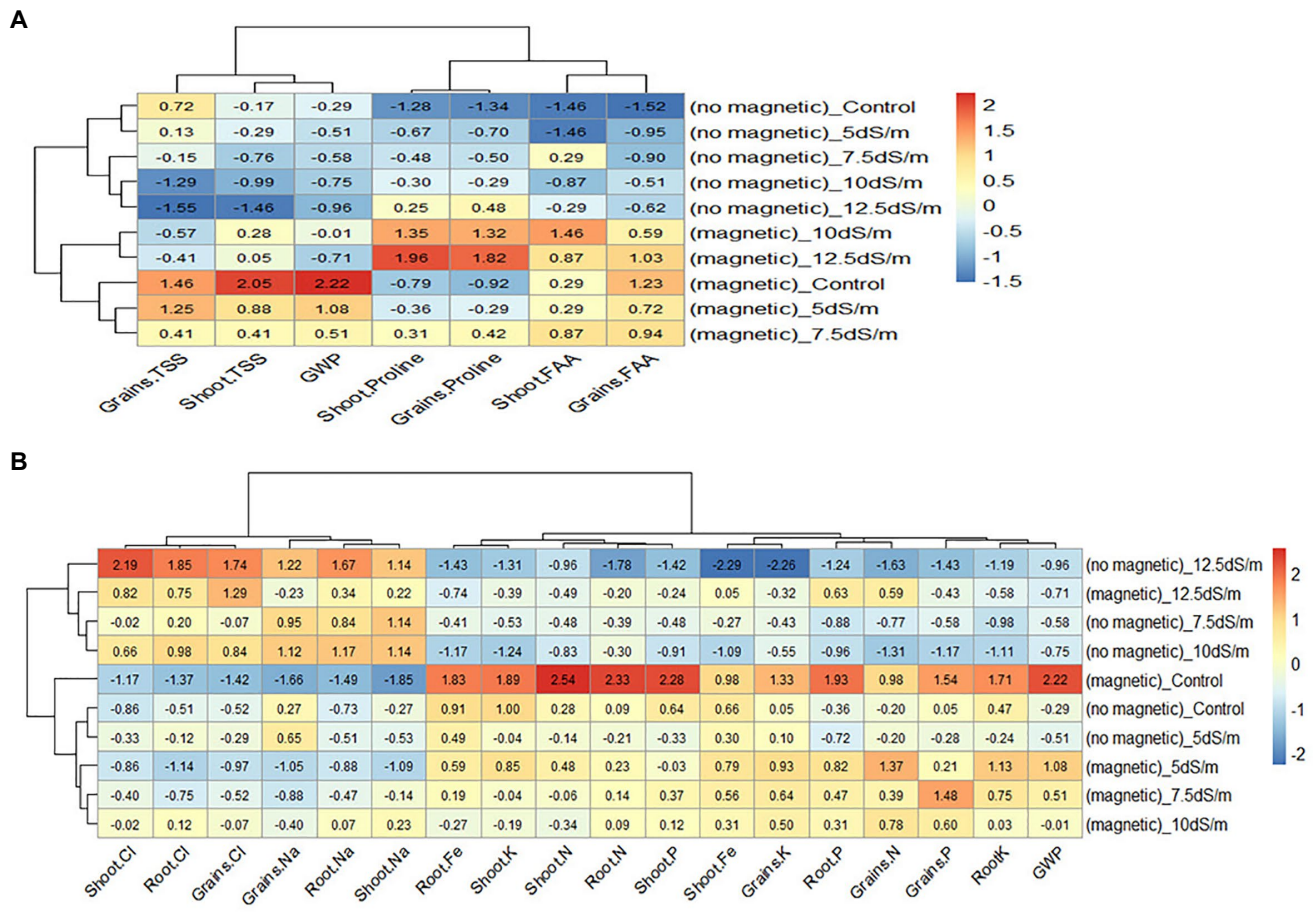
Heatmap of the relationship between the treatments and related parameters. The relationship between the treatments and the studied **(A)** traits and **(B)** nutrients. TSS, total soluble sugars; GWP, grain yield/plant; FAA, free amino acids.

Heatmap in Figure 4A showed the relationship between the treatments and the studied traits (TSS, FAA, and Pro in shoot and grains). The relationship was constructed based on standardized data using color scale. As the data were measured in different scales, they were standardized by subtracting the mean from each value and dividing by standard deviation. In the heatmap, cells with red color represents high values, while cells with blue color represents low values of the traits.

From the heatmap in Figure 4A, it was clear that high values of Pro (red color) in shoot and grains were found in the treatment with magnetic seawater 12.5 dS m^−1^ while the low values (blue color) were found in the treatment of no magnetic tap water. The highest values of TSS in shoot and grains were found in the treatment with magnetic tap water, while the lowest values were found in the treatment of no magnetic seawater 12.5 dS m^−1^. The lowest values of FAA were in no magnetic tap water, while the highest were in the treatment of magnetic seawater 10 dS m^−1^ for shoot and the treatment of magnetic tap water for grains. It seems that magnetizing seawater increased the Pro in both shoot and grains; however, wheat grain yield was more associated with TSS than FAA and Pro.

Heatmap in Figure 4B shows the relationship between the treatments and GWP, N, P, K, Na, and Cl in shoot, root, and grains as well as Fe in shoot and root. It is clear that GWP was inversely associated with Na and Cl in shoot, root, and grains; however, GWP was positively associated with N, P, and K in shoot, root, and grains as well as Fe in shoot and root.

In conclusion, magnetization was more effective under 5 and 7.5 dS m^−1^ than 10 and 12.5 dS m^−1^ where Na and Cl in shoot and root were decreased and consequently N, P, and K in both shoot and root were increased. These results may be due to the decreasing in water surface tension and viscosity which resulted in an improvement in water uptake (Takatshinko, 1997). Similarly, Elhindi et al. (2020) reported that the magnetizing water in pot marigold plant decreased Na and Cl, but increased N, P, and K.

## Discussion

Water scarcity and drought are increasingly significant environmental challenges impacting both plant growth and human uses. Furthermore, alternative water sources, such as recycled water, brackish water, seawater, and storm water, have been used for irrigation in various parts of the world and for a variety of purposes, including irrigating agricultural crops. Such examples from the current and previous studies showed the challenges associated with higher salinity water irrigation in general, and those which negatively affect wheat crops in particular (Qin and Horvath, 2020; Dolan et al., 2021).

The magnetic technology examined in this study was found to have a strong positive effect and alleviated the adverse effects of seawater irrigation on all the previously examined characteristics of wheat plant Sakha 93. In addition, the endogenous composition of the flag leaf and stem of wheat plants developed below seawater stress subjected to a magnetic field should be evaluated in comparison to the control.

Previous studies have found that irrigation with salt water considerably lowered the FW and DW of wheat and rice seedlings (Jasim et al., 2017). This negative effect of saline water on germination could be attributed to a decrease in leaf water potential, photosynthesis, and cell membrane degradation (Table 3; Supplementary Table S2). According to Parvanova et al. (2004), salt stress induces the formation of ROS, which causes oxidative damage to membrane lipids, proteins, and nucleic acids when available at high concentrations. This study found that at all seawater salinity levels, irrigation with magnetically treated seawater resulted in dramatic increases in all germination parameters compared with those in non-magnetic treatments.

Utilizing magnetically treated seawater increased the germination index and VI by approximately 49% and 31%, respectively, compared to untreated seeds under 7.5 dS m^−1^ salinity stress. Similar findings in Neva (poplar trees) where the irrigation with magnetically treated salt water boosted relative growth rate and leaf area of seedlings compared to non-magnetically treated salt water irrigation (Liu et al., 2019). This could be attributed to the effects of magnetic energy, which raise TWC and nutrient absorption (N, P, K, and Fe) through increases in the pH and reductions in the EC (Takatshinko, 1997; Tables 3 and 5). This effect resulted in improvements in the photosynthesis process and increases in products transfer in seedlings.

Turhan et al. (2014) have also reported similar results, demonstrating a substantial decrease in lettuce growth and DM when salt concentrations increased in irrigation water. When comparing low and high seawater levels, it was found that diluted seawater promoted *Conocarpus erectus* growth (El-Mahrouk et al., 2010). This finding may result from the fact that salinity inhibits photosynthesis, respiration, and protein synthesis, as well as causing higher osmotic pressure, lack of absorbed water, and harmful salt and chloride accumulation (Ferrante et al., 2011). Thus, all growth characteristics of wheat plants irrigated with magnetic seawater increased significantly.

Surendran et al. (2016) have observed that magnetic treatment of normal and saline water improved cow pea growth parameters. The positive effect of magnetism on plant growth characteristics may be attributed to the lower water surface tension and solubility (Takatshinko, 1997), and faster enzyme and hormone synthesis during the growth process (Selim et al., 2019). Thus, this may result in improved nutrient mobilization and transportation, and enhancing cell enlargement and vegetative growth.

Our results in Table 3 were consistent with those of Tuna et al. (2008), who previously reported that RWC was decreased when salinity levels increased in maize plants. The deleterious effect of seawater on the water relations and membrane integrity in wheat plants could possibly be due to the accumulation of toxic ions in leaves to harmful levels; thus, resulting in a significant reduction in water uptake which then leads to slower growth (Munns, 2002). Under salt stress, closing stomata, thickening of the leaf blade and epidermal cell diameters, increasing leaf mass per unit area, and rolling leaves, all reduce water transpiration and intracellular CO_2_ levels (Koundouras et al., 2008). The magnetic water irrigation in the second season of the current study; however, resulted in a substantial increase (*p* < 0.05) in TWC, osmotic pressure and MI; while LWD and TR were both reduced by approximately 12% and 16%, respectively, when irrigated with 10 dS m^−1^ seawater. According to our data, the obtained results in both seasons were almost similar in this study. These findings were in-line with Liu et al. (2019) of which magnetically treated water and magnetically treated saline water reduced TR when compared to controls. Lower surface tension, higher permeability, and lower hydrogen ions (Takatshinko, 1997), as well as an increase in the number and thickness of vessels in plant leaves and stems, might explain the observed improvements in water relations under seawater stress when utilizing magnetically treated water (Selim et al., 2019).

Salt stress reduced the Chl content of maize plants (Tuna et al., 2008); thus, it was consistent with our findings in the present study (Supplementary Table S1). The concentration of Na^+^ in seawater can damage the membrane system, decrease cell osmotic potential, stomatal conductance, gas exchange, and the absorption of iron ions. This is involved in Chl-protein production; and therefore, this resulted in a decrease in the photosynthetic rate (Tuna et al., 2008; Supplementary Table S2; Table 5). The use of magnetically treated seawater, on the other hand, resulted in a considerable increase in photosynthetic pigments in wheat plant leaves. In magnetically treated seawater of 12.5 dS m^−1^, the increase in total Chl *a* + *b*, Car, and UVAS was approximately 25%, 38%, and 33%, respectively, compared to the controls in the first season. The same trend was also observed during the second season. Liu et al. (2019) have shown that both magnetic tap water and saline water can improve the net photosynthetic rate of Neva plants when compared to the control. The enhancing effects of magnetically treated water could be due to the increases in protein production, enzyme activity, K^+^ levels and GA_3_ concentrations (Selim et al., 2009).

Our findings illustrated in Table 4 on wheat match those of Amuthavalli et al. (2012) when NaCl treatment can reduce the total sugar concentration in cotton (*Gossypium*). In their study, it was found that cotton plants synthesized Pro, aspartic acids, stress-related proteins, and enzyme levels using N ions when were under salinity stress. The patterns of grain measurements were similar to those examined in this study. These findings were consistent with those of Selim et al. (2019), claiming that the soluble sugar, total free amino acids, and Pro levels of wheat plants improved after magnetic treatments. Because soluble sugars and Pro accumulation can operate as osmoregulators, which stabilize cellular membranes and sustain turgor in response to increased salt levels, such increases could be a defense mechanism resulting in elevated osmotic pressure.

Tuna et al. (2008) have found that salt stress increased the antioxidant enzyme activity of peroxidase and polyphenol in maize plants, which normally protects them from the detrimental effects of activated oxygen species and salinity stress. Magnetic water at 12.5 dS m^−1^ was found to boost phenoloxidase and peroxidase enzyme activity in wheat plant leaves by approximately 24% and 26%, respectively, compared to the controls. Similarly, magnetic treatments increased the activity of the enzymes phenoloxidase and peroxidase in potato plants (Selim, 2019). When seawater levels elevated, macro- and micro-minerals in wheat plants and grains declined. Similarly, the treatment of high seawater levels decreased N, P, K, and Fe contents in spinach compared to that of low seawater levels (Turhan et al., 2014). On the other hand, irrigation with seawater caused a significant increase in Na content, resulting in an increase in the Na/K ratio in spinach plants. Irrigation with magnetically treated water enhanced the mineral contents of N, P, K, Fe, Zn, and Cu, but decreased Na^+^ and Cl^−^ levels in *Calendula officinalis* leaves (Elhindi et al., 2020). The reduction in the surface tension and viscosity of water, which increases the absorption of water and nutrients, as well as affecting many biosynthetic processes, could be related to the favorable effects of magnetically treated water (Takatshinko, 1997).

Salt stress has been shown to reduce plant hormones involved in cellular division, which has a negative impact on plant growth (Munns, 2002). Using magnetic technology, on the other hand, resulted in a considerable increase in the plant phytohormone concentrations in wheat plant shoots. Under seawater levels of 10 dS m^−1^, the concentrations of IAA, CKs and GA_3_ increased by approximately 6%, 62%, and 114%, respectively; whereas ABA decreased by 69%. These findings were aligned with those of Mousa et al. (2013) in wheat plants cultivated under salt soil stress, when magnetic treatments increased the concentrations of IAA, kinetin (CK) and GA_3_ but decreased the concentration of ABA in the shoot. Similarly, Turhan et al. (2014) have demonstrated that irrigation with seawater may result in reduced yield in lettuce. This could be attributed to the role of seawater in reducing water absorption, necessary nutrients and photosynthetic processes associated with high osmotic pressure of saline water (Tuna et al., 2008) as well as the inhibition of growth hormones (Table 6). Thus, this could have an effect on suppressing the vegetative growth and yield in wheat plant.

Our results were also consistent with what was found previously in potato (Mostafa, 2020) and cow pea (Surendran et al., 2016) plants, when the electromagnetic treatment of saline water has led to lessen the detrimental effects of salt while simultaneously increasing yield. This might be due to the fact of the involvement of magnetic water under seawater stress in enhancing photosynthetic pigments, water intake, nutrient availability, osmoregulatory chemicals, enzyme activity, and growth regulators. The physical and chemical features of magnetically treated water, including factors such as surface tension, polarity, hydrogen bonding, conductivity, salt solubility and pH, may be responsible for the observed improvements in physiological, growth, and yield factors (Takatshinko, 1997).

It has been reported that diluted saltwater up to 30% enhanced most stem and leaf structural parameters (El-Mahrouk et al., 2010). Similar results were also observed in the current study. Furthermore, seawater has been shown to inhibit plant hormone regulation of cellular division, which has a negative impact on plant growth (Munns, 2002). Moreover, the decrease in DNA content and CKs has led to the inhibition of cambial cell activity; consequently, reduced cell division and expansion as well as reduced leaf and stem diameters (Matsumoto-Kitano et al., 2008). Aligned with the magnetic approach used, Mousa et al. (2013) have revealed increased thickness of stems, leaves, metaxylem arteries, and vascular bundles in plants exposed to salt soil stress. Magnetic water increased the plant phytohormones (IAA, CKs, and GA_3_; Table 6), water uptake (Table 3), and elements absorption (Table 5), which enhanced the photoassimilate translocation and improved anatomical parameters (Aloni, 1995).

## Conclusion

The germination, growth, physiological, biochemical, and morphological features of the wheat cultivar (Sakha 93) were all negatively affected by irrigation with normal (non-magnetic) seawater. Magnetic technology, on the other hand, alleviated the negative effects of seawater stress on all the characteristics evaluated. Consequently, the GWP of wheat plants increased by approximately three-fold compared to the controls after applying a magnetic field. Therefore, the use of magnetically treated seawater of up to a level of 7.5 dS m^−1^ for wheat irrigation is recommended to replace the fresh tap water irrigation.

## Data availability statement

The original contributions presented in the study are included in the article/Supplementary material, and further inquiries can be directed to the corresponding authors.

## Author contributions

DS, MZ, KE-T, SA, and SE conceived and designed the research. ME-S, KE-T, SA, and SE supervised the study. DS, MA, HE, AE-T, and OI performed field experiments. DS, MZ, MA, ME-S, AE-T, and OI developed the biochemical and physiological analyses. DS, MB, KE-T, SA, and SE analyzed the data. MB and ME-S assisted with experiments and/or data evaluation. DS, KE-T, and SA wrote the manuscript. All authors contributed to the article and approved the submitted version.

## Funding

This project was funded by the Abu Dhabi Research Award (AARE2019) for Research Excellence-Department of Education and Knowledge (ADEK; grant number 21S105) to KE-T and Khalifa Center for Biotechnology and Genetic Engineering-UAEU (grant number 31R286) to SA.

## Conflict of interest

The authors declare that the research was conducted in the absence of any commercial or financial relationships that could be construed as a potential conflict of interest.

## Publisher’s note

All claims expressed in this article are solely those of the authors and do not necessarily represent those of their affiliated organizations, or those of the publisher, the editors and the reviewers. Any product that may be evaluated in this article, or claim that may be made by its manufacturer, is not guaranteed or endorsed by the publisher.

## Supplementary material

The Supplementary materials for this article can be found online at: https://www.frontiersin.org/articles/10.3389/fpls.2022.923872/full#supplementary-material
