# Supplementary material for: Germination, physio-anatomical behavior, and productivity of wheat plants irrigated with magnetically treated seawater
Source: Front Plant Sci. 2022 Aug 17;13:923872. doi: 10.3389/fpls.2022.923872 (PMC9431559; doi:10.3389/fpls.2022.923872)
Supplement: Supplementary file 1 [file Data_Sheet_1.pdf]

Supplementary material

Germination, physio-anatomical behavior, and productivity of wheat plants irrigated with magnetically treated seawater

Dalia A.-F.H. Selim, Muhammad Zayed, Maha M.E. Ali, Heba S. Eldesouky, Mercedes Bonfill, Amira M. El-Tahan, Omar M. Ibrahim, Mohamed T. El-Saadony, Khaled A. El-Tarabily*, Synan F. AbuQamar* and Samira ElOkkiah

*** Correspondence:**
Khaled El-Tarabily: ktarabily@uaeu.ac.ae
Synan AbuQamar: sabuqamar@uaeu.ac.ae

Supplementary material

Supplementary Table S1. Physical and chemical properties of the clay loam soil used.

Supplementary Table S2. Effect of seawater stress levels, magnetic treatment on the concentrations and the ratios of the photosynthetic pigment in leaves of wheat plants during the 1st and 2nd growing seasons.

Supplementary Table S1. Physical and chemical properties of the clay loam soil used.

Particle size distribution < 2 mm %					pH	EC_e dS.m dS.m^{-1}; 25°C	Soil paste extract analysis (meq.L^{-1})							
Coarse sand	Fine sand	Silt	Clay	Texture grade			Anions				Cations			
							$CO^{=}_2$	HCO^{-}_3	Cl^-	$SO^{=}_4$	Ca^{++}	Mg^{++}	Na^+	K^+
2.35	41.00	29.00	27.70	Clay loam	7.80	2.50	-	4.50	8.25	19.28	14.36	6.49	10.85	0.37

Supplementary Table S2. Effect of seawater stress levels, magnetic treatment on the concentrations and the ratios of the photosynthetic pigment in leaves of wheat plants during the 1st and 2nd growing seasons.

	Characteristics	Chl *a*	Chl *b*	Total Chl *a*+*b*	Car	Chl *a*/*b*	Total Chl/Car	UVAS
Magnetic Treatments	dS.m^{-1}	mg.g DW^{-1}						
First season								
No magnetic	Control	5.269^{abcd}	2.788^{ab}	8.057^{abc}	2.928^{ab}	1.909^{a}	2.753^{c}	2.676^{e}
	5	4.746^{bcde}	2.225^{cd}	6.971^{cde}	2.340^{abc}	2.142^{a}	3.001^{c}	2.763^{e}
	7.5	4.498^{cde}	2.063^{e}	6.561^{def}	2.136^{abcd}	2.210^{a}	3.077^{c}	2.289^{g}
	10	4.350^{de}	1.714^{f}	6.064^{ef}	1.123^{d}	2.541^{a}	5.421^{a}	2.399^{f}
	12.5	3.946^{e}	1.591^{g}	5.537^{f}	1.344^{cd}	2.673^{a}	4.123^{b}	2.405^{f}
Magnetic water	Control	6.072^{a}	2.810^{a}	8.882^{a}	3.102^{a}	2.170^{a}	2.863^{c}	3.953^{a}
	5	5.770^{ab}	2.702^{b}	8.472^{ab}	2.873^{ab}	2.195^{a}	2.953^{c}	2.955^{d}
	7.5	5.508^{abc}	2.312^{c}	7.819^{abcd}	2.427^{ab}	2.417^{a}	3.242^{bc}	2.715^{e}
	10	5.260^{abcd}	2.149^{de}	7.409^{abcd}	2.411^{ab}	2.477^{a}	3.077^{c}	3.405^{b}
	12.5	4.882^{bcde}	2.049^{e}	6.931^{cde}	1.850^{bcd}	2.385^{a}	3.756^{bc}	3.200^{c}
Second season								
No magnetic	Control	5.163^{b}	2.586^{c}	7.750^{bc}	2.784^{c}	1.996^{bc}	2.784^{de}	2.684^{a}
	5	4.498^{bcd}	2.063^{ef}	6.561^{cde}	2.386^{e}	2.210^{a}	2.749^{de}	2.770^{a}
	7.5	4.037^{cd}	2.114^{e}	6.150^{de}	2.251^{f}	1.915^{c}	2.736^{de}	2.234^{a}
	10	4.043^{cd}	1.985^{f}	6.028^{de}	2.002^{g}	2.036^{b}	3.015^{c}	2.422^{a}
	12.5	3.872^{d}	1.800^{g}	5.673^{e}	1.496^{h}	2.217^{a}	3.840^{b}	2.378^{a}
Magnetic water	Control	6.149^{a}	3.629^{a}	9.778^{a}	3.653^{a}	1.695^{d}	2.677^{ef}	2.934^{ab}
	5	5.257^{ab}	3.045^{b}	8.302^{b}	3.200^{b}	1.750^{d}	2.603^{f}	2.932^{ab}
	7.5	4.940^{bc}	2.440^{d}	7.381^{bcd}	2.600^{d}	2.025^{b}	2.840^{d}	2.672^{a}
	10	4.365^{bcd}	2.461^{d}	6.826^{cde}	2.410^{e}	1.775^{d}	2.835^{d}	3.314^{d}
	12.5	4.265^{bcd}	2.131^{e}	6.397^{cde}	1.961^{g}	2.007^{bc}	3.262^{b}	3.162^{b}

Values followed by different letters within a column are significantly different ($P < 0.05$).
Chl *a*, chlorophyll *a*; Chl *b*, chlorophyll *b*; Car, careteniods; UVAS; UV-absorbing substances.
